# Occurrence of 25 pharmaceuticals in Taihu Lake and their removal from two urban drinking water treatment plants and a constructed wetland

**DOI:** 10.1007/s11356-017-8830-y

**Published:** 2017-05-06

**Authors:** Xia-Lin Hu, Yi-Fan Bao, Jun-Jian Hu, You-Yu Liu, Da-Qiang Yin

**Affiliations:** Key Laboratory of Yangtze River Water Environment, Ministry of Education, College of Environmental Science and Engineering, Tongji University, 1239 Siping Road, Shanghai, 200092 China

**Keywords:** Pharmaceuticals, Drinking water, Water supply sources, Drinking water treatment plants, Constructed wetlands, Removal of contaminants

## Abstract

Pharmaceuticals in drinking water sources have raised significant concerns due to their persistent input and potential human health risks. The seasonal occurrence of 25 pharmaceuticals including 23 antibiotics, paracetamol (PAR), and carbamazepine (CMZ) in Taihu Lake was investigated; meanwhile, the distribution and removal of these pharmaceuticals in two drinking water treatment plants (DWTPs) and a constructed wetland were evaluated. A high detection frequency (>70%) in the Taihu Lake was observed for nearly all the 25 pharmaceutics. Chlortetracycline (234.7 ng L^−1^), chloramphenicol (27.1 ng L^−1^), erythromycin (72.6 ng L^−1^), PAR (71.7 ng L^−1^), and CMZP (23.6 ng L^−1^) are compounds with both a high detection frequency (100%) and the highest concentrations, suggesting their wide use in the Taihu Basin. Higher concentrations of chloramphenicols, macrolides, PAR, and CMZP were observed in dry season than in wet season, probably due to the low flow conditions of the lake in winter and the properties of pharmaceuticals. The overall contamination levels of antibiotic pharmaceutics (0.2–74.9 ng L^−1^) in the Taihu Lake were lower than or comparable to those reported worldwide. However, for nonantibiotic pharmaceutics, PAR (45.0 ng L^−1^) and CMZP (14.5 ng L^−1^), significantly higher concentrations were observed in the Taihu Lake than at a global scale. High detection frequencies of 25 pharmaceuticals were observed in both the two DWTPs (100%) and the wetland (>60%) except for florfenicol and sulfapyridine. The removal efficacies of the studied pharmaceuticals in DWTP B with advanced treatment processes including ozonation and granular activated carbon filtration (16.7–100%) were superior to DWTP A with conventional treatment processes (2.9–100%), except for sulfonamides. Wetland C with the constructed root channel technology was efficient (24.2–100%) for removing most pharmaceuticals. This work suggests that the application of cost-effective technologies such as constructed wetlands should be considered as an efficient alternative for removing pharmaceuticals from water supply sources.

**Electronic supplementary material:**

The online version of this article (doi:10.1007/s11356-017-8830-y) contains supplementary material, which is available to authorized users.

## Introduction

Pharmaceuticals are any synthesized chemical compounds or drugs designed to cure and prevent the spread of diseases as well as adding value to humans and animals life (Tijani et al. 2013). Pharmaceuticals include antibiotics, antiepileptic, antimicrobials, and antianxiety medications. The rampant usage of pharmaceuticals has made their occurrence ubiquitous in the environment. Pharmaceuticals in the environment have received increased attention over the past decade. The contamination of pharmaceuticals has been reported in both natural and artificial systems, including soil, sediment, sludge, groundwater, wastewater, tap water, surface water, and aquatic organisms (Gothwal and Shashidhar 2015). Pharmaceutical concentrations in the natural aquatic environment are in the ranges of sub-ng to low μg L^−1^, well below acute toxic levels. However, there are uncertainties regarding the effects of chronic exposures (Rand-Weaver et al. 2013). The adverse effects on aquatic communities include the feminization of male fish (Corcoran et al. 2010), development of pathogen resistance (Zuccato et al. 2006), and decrease in plankton diversity (Farré et al. 2008).

Wastewater treatment plants (WWTPs) are regarded as one of the most important sources of pharmaceuticals in the environment. Occurrence and removal of pharmaceuticals in wastewaters and municipal WWTPs has been widely studied. No specific pattern is observed in the occurrence of pharmaceuticals in wastewaters (Evgenidou et al. 2015). Great discrepancies are observed between countries indicating the regional differences in drug use patterns (Luo et al. 2014; Mailler et al. 2016). Variations are also observed within the same country, between different WWTPs (Guerra et al. 2014), probably due to the different pharmaceutical prescription patterns in a country and the varied removal efficiencies of individual WWTPs. The conventional sewage treatment facilities were never specially designed to deal with pharmaceuticals; it is found that most pharmaceuticals cannot be fully removed (Michael et al. 2013). Due to their highly variable physicochemical properties (chemical structure, octanol/water partition coefficient) as well as the operational conditions of the biological process, the efficiencies by which pharmaceuticals are removed vary substantially (Michael et al. 2013; Evgenidou et al. 2015). The efficiency of WWTPs for the elimination of pharmaceuticals is under the control of operational conditions (the hydraulic retention time, sludge retention time, and biodegradation kinetics) and environmental conditions (the temperature, redox conditions, and pH) (Evgenidou et al. 2015). Regarding the physicochemical characteristics of the pharmaceuticals that affect their removal by WWTP’s processes, the ability to interact with solid particles is a major factor because it facilitates their removal by physical–chemical (settling, flotation) or biological processes (biodegradation). Compounds with low adsorption coefficients tend to remain in the aqueous phase, which favors their mobility through the WWTP and into the receiving waters. Advanced treatment processes, such as activated carbon adsorption and advanced oxidation processes can achieve higher and more consistent removal efficiencies for micropollutants (Luo et al. 2014).

Drinking water is one of the most important sources for uptake of pharmaceuticals by human beings. Several studies were carried out to investigate the occurrence and behavior of pharmaceuticals in drinking water sources and drinking water treatment plants (DWTPs) (Qiao et al. 2011; de Jesus Gaffney et al. 2015; Simazaki et al. 2015). Conventional drinking water treatment methods such as coagulation, sedimentation, and filtration have poor removal efficiencies for most pharmaceuticals. The removal efficiencies of the advanced water treatment processes including ozonation and granular activated carbon filtration were typically much higher than those of the conventional treatment processes. Constructed wetlands could serve as a cost-effective and promising alternative to conventional wastewater treatment methods for removing pharmaceuticals in treated water (Hijosa-Valsero et al. 2010; Dordio and Carvalho 2013; Li et al. 2014; Verlicchi and Zambello 2014; Zhang et al. 2014). Constructed wetlands can facilitate the removal of pharmaceuticals via natural processes involving plants, microorganisms, solid matrix components, and sunlight (Dordio and Carvalho 2013). The mechanisms involved in pollutant removal in constructed wetlands can be classified into biotic processes (e.g., microbiological degradation, biofilm, root, and plant uptake) and physicochemical processes (evaporation, photodegradation, oxidation, hydrolysis, retention, or root sorption into the gravel bed) (Verlicchi and Zambello 2014; Zhang et al. 2014; Hijosa-Valsero et al. 2016). The removal efficiency of pharmaceuticals in constructed wetlands has been demonstrated to be comparable to conventional wastewater treatment processes (Matamoros et al. 2008; Verlicchi and Zambello 2014). Therefore, constructed wetlands might be also promising for the treatment of source water prior to DWTPs. However, as to our knowledge, no available studies investigate the occurrence and removal of pharmaceuticals in constructed wetlands for pretreating source water. The comparison of the removal efficiency between constructed wetlands and drinking water treatment processes (both conventional and advanced treatment processes) is also needed.

Taihu Basin, one of the highly developed and densely populated regions in China, is situated in the southeastern Yangtze River Delta of the East China. Taihu Lake is the third largest freshwater lake in China, with an average water depth of 1.9 m and a surface water area of 2338 km^2^. The lake serves as an important entertainment and tourist attraction. The lake water is used for agricultural and industrial purposes, and it is also the source of drinking water for Shanghai, Suzhou, and Wuxi. Along with the rapid development of the economy and the intensive use of water resources, the lake is getting more and more seriously polluted by various pollutants, including heavy metals and persistent organic pollutant (Wang et al. 2011; Yu et al. 2014). However, the occurrence of emerging pollutants such as pharmaceuticals in Taihu Lake has been scarcely studied (Bu et al. 2013; Xu et al. 2014; Xie et al. 2015), especially the seasonal occurrence. Previous work (Xu et al. 2014; Xie et al. 2015) only investigated a limited number of pharmaceuticals (<15) in Taihu Lake, and chloramphenicols were never included. Also, the distribution of antibiotic pharmaceutics and nonantibiotic pharmaceutics were not compared. Most of all, there was no information about seasonal variation of these pharmaceuticals in Taihu Lake. Therefore, it is significant to investigate the seasonal occurrence of multiclass pharmaceuticals in the Taihu Lake.

The objective of this paper is to investigate the occurrence and seasonal variations of 25 pharmaceuticals including 23 antibiotics (four tetracyclines, three chloramphenicols, six sulfonamides and trimethoprim, six fluoroquinolones, three macrolides), an antipyretic analgesic (paracetamol), and an antiepileptic drug (carbamazepine) in Taihu Lake. The occurrence and removal efficiencies of pharmaceuticals in two drinking water treatment plants and a constructed wetland for pretreating source water is also investigated and compared. The results will provide important background data for risk assessment and pollutant control. The comparison among different drinking water treatment techniques will also plot an overview of currently used techniques for pharmaceuticals’ removal in this area.

## Materials and methods

### Study areas and sampling site selection of Taihu Lake

**Fig. 1:**
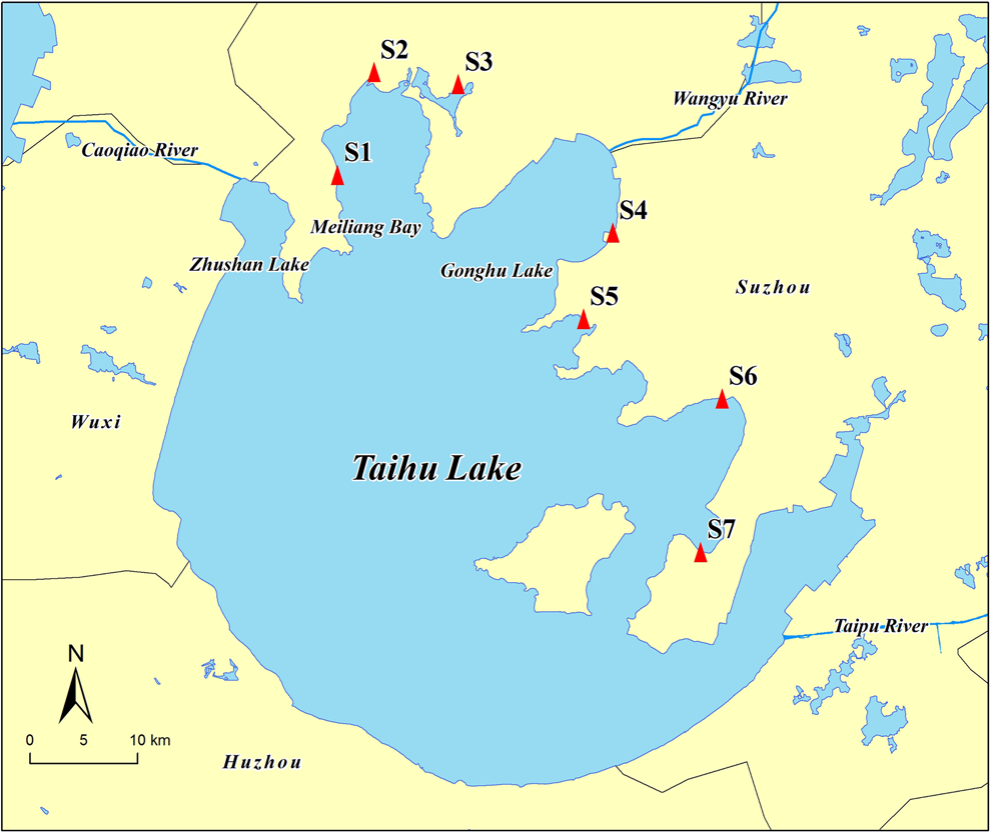
Sampling sites in northeastern Taihu Lake

The sampling sites were set in several key districts in the northeastern part of Taihu Lake including mainly Wuxi Taihu and Suzhou Taihu. A total of seven sampling sites were chosen, including three sites in the highly polluted Meiliang Bay of Wuxi (S1: north latitude 31° 28′ 14.45″ N, east longitude 120° 07′ 56.43″ E; S2: north latitude 31° 33′ 1.12″ N, east longitude 120° 11′ 13.75″ E; S3: north latitude 31° 31′ 34.96″ N, east longitude 120° 16′ 1.15″ E), a site in Wuxi Gong Bay (S4: north latitude 31° 22′ 38.13″ N, east longitude 120° 23′ 18.92″ E), and three sites in Suzhou Taihu (S5: north latitude 31° 18′ 37″ N, east longitude 120° 20′ 37″ E; S6: north latitude 31° 13′ 15.76″ N, east longitude 120° 27′ 45.95″ E; S7: north latitude 31° 05′ 48.54″ N, east longitude 120° 24′ 44.21″ E). Their locations are shown in Fig. 1. Two sampling events were conducted in June and December, 2015. In June 2015, the rainfall was 332.7 mm and the average water level reached 3.56 m. In December 2015, the rainfall was 60.1 mm and the water level was 3.46 m (http://www.tba.gov.cn//tba/, in Chinese).

### Two drinking water treatment plants and the constructed wetland

**Fig. 2:**
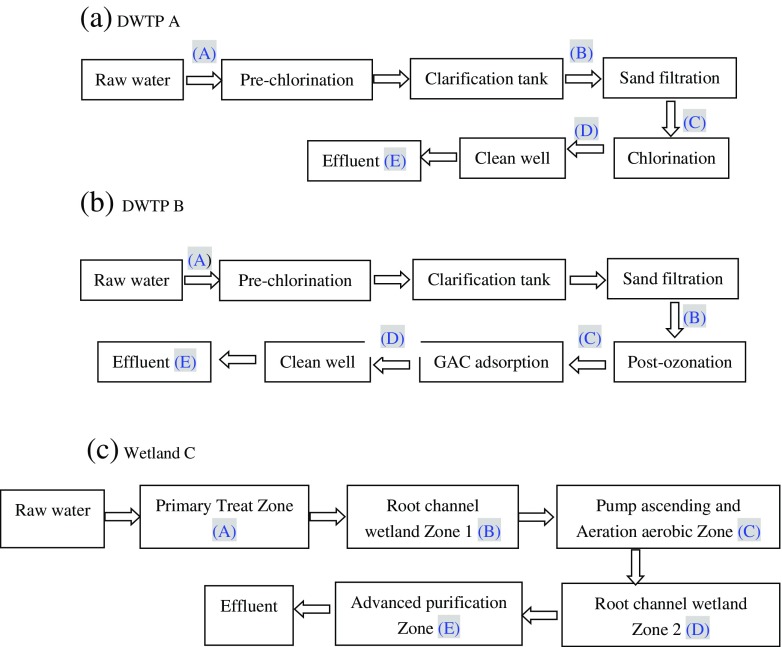
The processes and sampling sites in the two drinking water treatment plants (DWTPs) and the constructed wetland. **a** DWTP A. **b** DWTP B. **c** Wetland C

Two typical DWTPs, one employing the conventional treatment process (A) and the other employing the conventional treatment process followed by an advanced treatment process (B), were chosen. These two DWTPs share the same source water from the Taihu Basin. DWTP A employs the conventional treatment process, which treats raw water with chlorination-coagulation/flocculation–clarification–filtration–chlorination sequentially. DWTP B uses an advanced treatment process following the conventional treatment process, which treats raw water with chlorination-coagulation/flocculation–clarification–filtration–postozonation–granular activated carbon (GAC) adsorption sequentially. Figure 2a, b shows the main processes of each DWTP and sampling.

The constructed wetland (C) is currently the largest drinking source water treatment wetland system in China. It provides purified raw water for the drinking water plant in Jiaxing City, Taihu Basin. Wetland C mainly uses the constructed root channel technology (CRCT) and multilevel ponds/plant bed-ditch system synergic purification technology as the core technologies to purify the micropolluted water source (Zheng et al. 2012). Figure 2c shows the main zones of the wetland, including bio-pretreatment zone, root channel wetland ecological purification zone, ecological purification zone, and advanced purification zone. Different plants are grown in different zones, depending on the functions of each zone.

### Sample collection

The water samples from the Taihu Lake were collected twice in June and December 2015. The water samples from the two DWTPs and the constructed wetland were collected in June 2015. The water samples (4 L per site) were collected with a clean stainless steel sampler, and the sampler and precleaned brown bottles were washed three times with the target water sample. The samples were stored at 4 °C to prevent degradation. Water samples were extracted within 2 days from the time of collection.

### Chemicals, sample preparation, and analysis

All standards of pharmaceuticals and chemicals used in sample preparation and analysis were of HPLC grade or analytical grade. Water samples were enriched by the solid-phase extraction (SPE) using the Oasis HLB cartridges (500 mg, 6 mL, Waters, Millford, MA, USA). Before extraction, water samples were filtered through 0.45-μm glass fiber filters (GF/F, Whatman, Mainstone, England), and then acidified to pH 3.0 with formic acid, followed by adding 0.5 g L^−1^ Na_2_EDTA as the chelating agent. Finally, isotopically labeled internal standards, including sulfamethazine-^13^C_6_, sulfamethoxazole-^13^C_6_, ciprofloxacin (^13^C_3_^15^N), erythrocin-^13^C_2_, and trimethoprim-D_3_, were added in the samples with final concentrations at 25 ng L^−1^. Details about SPE cleanup are described in Supplementary material. The pharmaceutical analysis was performed by ultra performance liquid chromatography-tandem mass spectrometry (UPLC-MS/MS) (Waters Xevo TQ MS, Milford, MA, USA). All 25 pharmaceuticals were separated by a 150 mm BEH C_18_ column. Mass spectrometer detections of pharmaceuticals were operated in selected reaction monitoring mode. The details of separation and mass spectrometer parameters for each compound are described in Table S1 (Supplementary material).

Quantitative standard curve was obtained by plotting the ratio of the compound peak area with the internal standard peak area versus their concentrations. A series of mixed standard solutions were prepared (0, 0.1, 0.2, 0.5, 1, 2, 5, 10, 20, 50, 100, and 200 ng L^−1^) to evaluate the linearity. The analytical performance of the SPE-HPLC-MS/MS method was satisfying for target compounds, with wide liner ranges (0.1–200 ng L^−1^) and good correlation coefficients (*R* ^2^ > 0.99). The limits of detection (LODs) and limits of quantification (LOQs) of 25 pharmaceuticals in surface water ranged from 0.12 to 2.24 ng L^−1^ and from 0.41 to 7.48 ng L^−1^, respectively. The recoveries of 25 pharmaceuticals (25 and 50 ng L^−1^) spiked to filtered surface water samples were mostly in the range of 70 to 110% (*n* = 3). The intra-day relative standard deviation (RSD) (*n* = 3) and inter-day RSD (*n* = 6) in the surface water was less than 15 and 25%, respectively. Analysis of reagent blanks demonstrated that the analytical system and glassware were free of contamination. The detailed information is shown in Table S2 (Supplementary material).

## Results and discussion

### Occurrence and seasonal variation of pharmaceuticals in Taihu Lake

#### Overview of the occurrence: detection frequencies and levels of concentrations

Most of the 25 pharmaceuticals were detected at all sampling sites in both sampling events. The results including the average, median, maximum, and minimal concentrations in all the sampling sites, as well as detection frequencies are summarized in Table 1. A highest detection frequency of 100% is observed for 11 pharmaceuticals including four tetracyclines, CAP, SMR, TMP, FLE, ERY, PAR, and CMZP in both June and December at all the seven sampling sites. This suggests the wide applications of these 11 pharmaceuticals in Taihu area. A lowest detection frequency (<20%) is observed for ROX in both June and December in Taihu Lake. The detection frequency of 21 pharmaceuticals is higher than 70% in the wet season (June), except for FF (29%), SPD (29%), ROX (14%), and TYL (14%). The detection frequency of 22 pharmaceuticals is 100% in the dry season (December), except for FF (17%), SPD (67%), and ROX (17%). From Table 1, we can also see that the number of compounds with a 100% detection frequency is 22 (accounting for 88% of the 25 compounds) and 12 (accounting for 48% of the 25 compounds) in dry season (December) and wet season (June), respectively. For all the individual pharmaceuticals except for FF, the detection frequency is higher in dry season (December) than that in wet season (June). Figure S1 shows that 18 to 25 compounds were detected in June, whereas 23 to 25 compounds were detected in December in each sampling site. For the same sampling site, a larger number of detected compounds were also observed in December than that in June. In general, the detection frequency of pharmaceuticals in Taihu Lake is higher in dry season than that in the wet season.

**Table 1:** Detection frequencies and concentrations of 25 pharmaceuticals in seven sampling sites of Taihu Lake

Compound		Log*K*ow^a^	pKa^a^ Strongest acidic/basic	Charge at pH 7.0	June, 2015					December 2015				
					Frequency^b^ (%)	Concentration (ng L^−1^)				Frequency (%)	Concentration (ng L^−1^)			
						Aver.^c^	Med.^d^	Max.^e^	Min.^f^		Ave.^c^	Med.^d^	Max.^e^	Min.^f^
Tetracyclines	Doxycycline (DOX)	−0.02	−2.2, 7.5	Negatively charged	100	5.8	5.0	8.3	3.2	100	19.4	15.6	44.0	9.3
	Tetracycline (TC)	−1.30	−2.2, 8.24	Negatively charged	100	20.1	16.9	39.7	12.7	100	17.1	11.9	40.9	10.3
	Oxytetracycline (OTC)	−0.99	0.24, 7.75	Negatively charged	100	16.2	14.1	30.6	8.3	100	20.9	18.5	31.9	14.8
	Chlortetracycline (CTC)	–	–	–	100	74.9	17.6	234.7	5.8	100	54.6	54.5	72.8	35.4
Chloramphenicols	Chloramphenicol (CAP)	1.14	7.49, −2.8	Neutral	100	17.6	16.4	27.1	9.7	100	47.5	45.9	65.5	27.6
	Thiamphenicol (TAP)	–	–		86	4.8	3.3	10.3	<LOD	100	2.9	2.7	5.6	1.2
	Florfenicol (FF)	−2.23	–		29	8.8	<LOD	10.1	<LOD	17	2.6	<LOD	2.6	<LOD
Sulfonamides	Sulfapyridine (SPD)	0.35	6.24, 2.63	Negatively Charged	29	0.2	<LOD	0.4	<LOD	67	0.3	0.3	0.4	<LOD
	Sulfadiazine (SD)	−0.09	6.99, 2.01	Negatively Charged	86	2.0	1.0	6.0	<LOD	100	5.1	4.8	7.2	3.3
	Sulfamethoxazole (SMX)	0.89	6.16, 1.97	Negatively Charged	86	8.2	8.4	12.4	<LOD	100	2.6	2.2	5.0	1.8
	Sulfamerazine (SMR)	0.14	6.99, 2.01	Negatively Charged	100	2.2	1.8	2.7	1.4	100	1.1	0.9	1.6	0.8
	Sulfamethazine (SMZ)	0.89	6.99, 2.04	Negatively Charged	86	1.8	0.8	6.5	<LOD	100	0.9	0.8	1.6	0.4
	Sulfachloropyridazine (SCP)	–	–		100	2.1	2.2	4.2	0.8	100	1.8	1.4	4.0	0.9
Trimethoprim (TMP)		0.91	17.33, 7.16	Positively charged	100	12.1	8.8	29.7	4.7	100	4.8	1.9	14.9	1.5
Fluoroquinolones	Norfloxacin (NOR)	−1.03	5.77, 8.68	Neutral	86	5.9	6.5	10.5	<LOD	100	11.0	11.1	13.2	9.2
	Ciprofloxacin (CIP)	0.28	5.76, 8.68	Neutral	86	5.2	2.4	16.6	1.9	100	4.3	4.5	6.3	2.0
	Enrofloxacin (ENR)	1.88			71	16.0	9.7	39.2	<LOD	100	7.1	4.6	23.2	2.1
	Ofloxacin (OFL)	−0.39	−2.4, 5.45	Negatively charged	71	2.9	2.4	5.7	<LOD	100	2.6	2.3	4.3	1.9
	Fleroxacin (FLE)	0.24	–	–	100	4.6	3.5	10.2	2.0	100	2.0	0.8	6.8	0.6
	Sarafloxacin (SAR)	2.5	–	–	71	3.5	2.7	8.3	<LOD	100	2.7	2.5	3.9	1.6
Macrolides	Erythromycin (ERY)	3.06	12.44, 8.38	Positively charged	100	38.6	35.5	72.6	12.1	100	44.7	42.6	67.5	24.9
	Roxithromycin (ROX)	1.7	12.45, 9.08	Positively charged	14	1.0	<LOD	1.0	<LOD	17	14.4	15.5	18.4	<LOD
	Tylosin (TYL)	3.41	7.1	Neutral	14	1.2	<LOD	1.2	<LOD	100	1.4	1.0	3.2	0.7
Paracetamol (PAR)		0.46	9.46, −4.4	Neutral	100	45.0	44.2	71.7	21.0	100	58.5	55.5	85.2	40.0
Carbamazepine (CMZP)		2.45	−5.96,-3.8	Neutral	100	14.5	12.7	23.6	6.3	100	17.4	17.7	28.5	7.1

*LOD* limit of detection

^a^Log*K*ow and pKa adopted from Drugbank

^b^Frequency of detection of each antibiotic in all water samples (*n* = 7)

^c^Average

^d^Median

^e^Maximum

^f^Minimal

Concentrations of all detected pharmaceuticals were typically in the ng L^−1^ level (Table 1). In June, the average concentrations were in the range of 0.2 to 74.9 ng L^−1^, the median concentrations were in the range of 0.8 to 35.5 ng L^−1^, and the maximum concentration was 234.7 ng L^−1^ (CTC, S1). In December, the average concentrations were in the range of 0.3 to 58.5 ng L^−1^, the median concentrations were in the range of 0.3 to 55.5 ng L^−1^, and the maximum concentration was 85.2 ng L^−1^ (PAR, S5). In June, CTC showed the highest average concentration (74.9 ng L^−1^) and maximum concentration (234.7 ng L^−1^); PAR showed the second highest detection concentrations (average concentration 45.0 ng L^−1^, maximum concentration 71.7 ng L^−1^). In December, PAR showed the highest detection concentrations (average concentration 55.5 ng L^−1^, maximum concentration 85.2 ng L^−1^), followed by CTC, with the average concentration of 54.6 ng L^−1^ and maximum concentration of 72.8 ng L^−1^. SPD showed the lowest concentrations in both June and December (maximum concentration 0.4 ng L^−1^). The highest concentrations of CTC and PAR suggest that these two compounds should be paid more attention to.

### Seasonal variation and spatial distribution

Tetracyclines are broad spectrum antibiotics and the second widely used human and/or veterinary antibiotics in the world (Bu et al. 2013). All four tetracyclines were detected at a frequency of 100%. In June, CTC (average 74.9 ng L^−1^, maximum 234.7 ng L^−1^) was found to be the compound with the highest concentration among all 25 analytes, followed by TC (average 20.1 ng L^−1^, maximum 39.7 ng L^−1^), OTC (average 16.2 ng L^−1^, maximum 30.6 ng L^−1^), and DOX (average 5.8 ng L^−1^, maximum 8.3 ng L^−1^). In December, highest average concentrations were also observed for CTC (54.6 ng L^−1^), followed by OTC (20.9 ng L^−1^), DOX (19.4 ng L^−1^), and TC (17.1 ng L^−1^). Nearly all studies selected TC and OTC as target contaminants in the survey of water contamination in China by tetracyclines, due to the fact that the two chemicals were once administrated in China for human and veterinary uses (Bu et al. 2013). The other two tetracyclines (CTC and DOX) were seldom reported in the natural water probably due to their strong absorption to sediments and particles as well as degradation (Sarmah et al. 2006; Bu et al. 2013). The concentration levels of tetracyclines in Taihu Lake were comparable to these in surface waters of China and the world (ND −1036 ng L^−1^) (Watkinson et al. 2009; Bu et al. 2013).

Chloramphenicols were detected at different frequencies and average concentrations in June and December. Only CAP was detected at 100% in both June and December. The highest average concentration was observed for CAP (17.6 ng L^−1^ in June, 47.5 ng L^−1^ in December), followed by FF (8.8 ng L^−1^ in June). Comparable average concentrations were found for FF (2.6 ng L^−1^ in December) and TAP (4.8 ng L^−1^ in June, 2.9 ng L^−1^ in December). The maximum concentrations of chloramphenicols were 65.5 ng L^−1^ (December), 10.3 ng L^−1^ (June), and 10.1 ng L^−1^ (June) for CAP, TAP, and FF, respectively. Chloramphenicols are seldom investigated in Chinese surface waters or throughout the world (Jiang et al. 2011; Bu et al. 2013; Yan et al. 2015a). The chloramphenicol values in Taihu Lake were comparable to or relatively lower than those in China and the world (ND −266 ng L^−1^) (Jiang et al. 2011; Bu et al. 2013; Yan et al. 2015a).

Sulfonamides and trimethoprim are most frequently reported antibiotics in surface water (Jiang et al. 2011; Bu et al. 2013). SMZ, SMX, and SD are the extensively studied sulfonamides in China due to their use in veterinary medicines (Bu et al. 2013). TMP was detected at a 100% frequency in both June and December, with the maximum concentration of 29.7 ng L^−1^ (June) and 14.9 ng L^−1^ (December). Two sulfonamides (SMR, SCP) in June and five sulfonamides (six out of SPD) in December were detected at a 100% frequency. Sulfonamides and trimethoprim have high potential to resist degradation and are hydrophilic enough (log*K*ow < 1, Table 1) to transport in the aquatic environment for a long distance. These properties might contribute to their high detection frequencies in the aquatic environment. The average concentrations of sulfonamides ranged from 0.2 ng L^−1^ (SPD) to 8.2 ng L^−1^ (SMX) in June and from 0.3 ng L^−1^ (SPD) to 5.1 ng L^−1^ (SD) in December. Maximum concentrations of all the six sulfonamides were below 10 ng L, except for SMX (12.4 in June). The concentrations of sulfonamides in Taihu Lake is comparable to or lower than that reported in other areas of China (ND −940 ng L^−1^) (Jiang et al. 2011; Bu et al. 2013; Wang et al. 2015) or throughout the world (Choi et al. 2008; Vulliet and Cren-Olivé 2011). Concentrations of most individual sulfonamide were related to the highly populated megacities such as Beijing, Shanghai, and Guangzhou in China. The intensive aquacultural and poultry–fishery activities could be other sources of sulfonamides (Bu et al. 2013).

Fluoroquinolones are the most important synthetic antibiotics produced and consumed as both human and veterinary medicines in China. Fluoroquinolones were detected at high frequencies ranging from 71 to 100%, of which FLE in June and all the six compounds in December were detected at a 100% frequency. The average concentrations ranged from 2.9 ng L^−1^ (OFL) to 16.0 ng L^−1^ (ENR) in June and 2.0 ng L^−1^ (FLE) to 11.0 ng L^−1^ L (NOR) in December. The maximum concentrations were observed for ENR both in June (39.2 ng L^−1^) and December (23.2 ng L^−1^). Significantly higher detection frequencies and remarkably higher concentrations of fluoroquinolones were found in our study than those reported in previous work (ND) (Jiang et al. 2011). However, these concentrations were comparable to or slightly lower than those reported in most studies conducted in other places throughout the world (ND −634 ng L^−1^) (Bu et al. 2013).

Macrolides were detected at average concentrations ranging from 1.0 (ROX) to 38.6 ng L^−1^ (ERY) in June, and 1.4 (TYL) to 44.7 ng L^−1^ (ERY) in December. A high detection frequency at 100% was observed for ERY in both June and December, whereas a detection frequency lower than 17% was observed for ROX in both June and December and TYL in June (14%). The higher concentrations of ERY might be due to the biodegradation-resistant of ERY (McArdell et al. 2003). PAR and CMZP were detected at average concentrations of 45.0 ng L^−1^ in June and 58.5 ng L^−1^ in December, as well as 14.5 ng L^−1^ in June and 17.4 ng L^−1^ in December. The detection frequency of PAR and CMZP was 100% in all the sampling events. Concentrations of macrolides in the Taihu Lake were much higher than those in the Huangpu River (Jiang et al. 2011) and lower than those in the Yangtze River, China (Wu et al. 2014). Comparable concentrations of PAR (41 ng L^−1^) in the Yangtze River were also observed by Wu et al. (2014). Comparable concentrations or lower levels of macrolides were observed in the Taihu Lake in a global scale (ND −3700 ng L^−1^) (Bu et al. 2013). However, significantly higher concentrations of PAR and CMZP were observed in the Taihu Lake in a global view (global PAR ND −230 ng L^−1^ and CMZP ND −8.05 ng L^−1^) (aus der Beek et al. 2016).

**Fig. 3:**
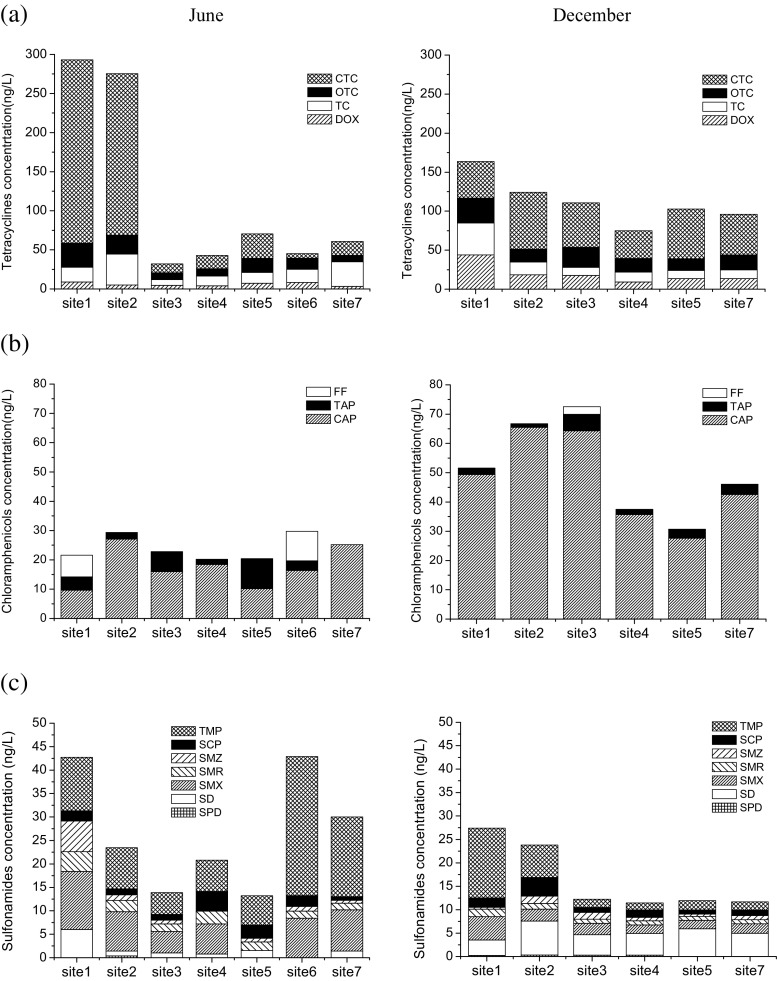

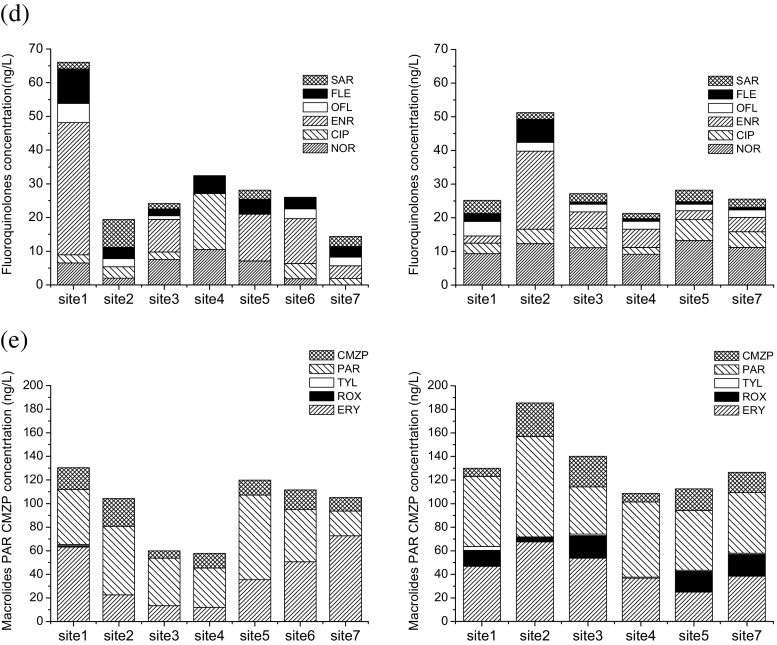
Seasonal and spatial distributions of 25 pharmaceuticals in Taihu Lake. **a** Tetracyclines. **b** Chloramphenicols. **c** Sulfonamides and trimethoprim. **d** Fluoroquinolones. **e** Macrolides, paracetamol, and carbamazepine

Generally, the overall contamination levels of antibiotic pharmaceutics in the Taihu Lake were lower than or comparable to those reported worldwide (Ferrer et al. 2010; Bu et al. 2013; Wu et al. 2014; Wang et al. 2015). However, for nonantibiotic pharmaceutics, PAR and CMZP, significantly higher concentrations were observed in the Taihu Lake in a global scale (aus der Beek et al. 2016). High detection frequencies (>70%) were observed for nearly all the 25 pharmaceutics except for FF, SPD, ROX, and TYL. Tetracyclines, CAP, ERY, PAR, and CMZP are compounds with both a high detection frequency (100%) and the highest concentrations, suggesting their wide use in the Taihu Basin. In general, the detection frequency of pharmaceuticals in Taihu Lake is higher in dry season than that in wet season. For chloramphenicols (Fig. 3b), macrolides, PAR, and CMZP (Fig. 3e), the overall pharmaceutical levels in dry season (December) were higher compared with those in wet season (June). The total concentrations of chloramphenicols at each sampling site were approximately 1.1 to 3.5 times greater in December than in June, of which CAP concentrations were 1.1 to 6.5 times greater in December than in June. The total concentrations of macrolides were 0.9 to 6.1 times greater in December than in June. The mean concentration of ROX in December especially increased 19.2 times compared with that in June, which was the highest ratio among all target compounds. However, sulfonamides (Fig. 3c) were observed higher concentrations in wet season (June); tetracyclines (Fig. 3a) and fluoroquinolones (Fig. 3d) showed no obvious trends. Lower concentrations of chloramphenicols, macrolides, PAR, and CMZP in June than that in December might be easily explained by the flow conditions of the lake and the properties of pharmaceuticals. The high flow conditions in June might result in a great dilution on the concentrations of pharmaceuticals in the surface water. Biodegradation and photodegradation of pharmaceutics might be higher in summer than that in winter due to the higher activity of microorganism and strong sunlight of summer (Jiang et al. 2011). Also, PAR is mainly used for curing a cold, of which the high incidence is winter. It is also noteworthy that chloramphenicols, macrolides, PAR, and CMZP are compounds with relatively higher hydrophobicity among all the 25 pharmaceuticals (Table 1). These compounds might be strongly adsorbed to sediments and solids due to their higher hydrophobicity (Xu et al. 2014). However, they might be prone to be desorbed from the sediments and solid to the surface water in winter due to the low water level, resulting in higher concentrations in dry season than that in wet season. Future work focusing on adsorption of pharmaceuticals in sediments and partition between surface water and sediments is needed. Also, further work including more sampling events would be required to give a definitive seasonality.

The spatial distribution of pharmaceuticals in the Taihu Lake is shown in Fig. 3. It was found that the highest concentrations of nearly all kinds of pharmaceuticals were always found in S1 and S2. The highest total concentrations were observed at 293.1 ng L^−1^ (S1) and 275.4 ng L^−1^ (S2) for tetracyclines, 50.13 ng L^−1^ (S1) and 42.9 ng L^−1^ (S6) for sulfonamides, 129.27 ng L^−1^ (S1) for fluoroquinolones, as well as 65.4 ng L^−1^ (S1) and 72.6 ng L^−1^ (S7) for macrolides. However, chloramphenicols, PAR, and CMZP showed no obvious trends among all the seven sampling sites. Also, largest numbers of detected compounds were observed in S1 (25) and S2 (21). Highest concentrations of total antibiotics (15 kinds) were also observed in the Northern Taihu by Xu et al. (2014). The reason might be the high pollution of Meiliang Bay (S1 and S2) in the northern Taihu Lake, due to the dense population and advanced economy around Suzhou and Wuxi City.

### Occurrence and removal of pharmaceuticals in two DWTPs and a constructed wetland

#### Overview of the occurrence: detection frequencies and levels of concentrations

As shown in Table S3, pharmaceuticals were detected in both the two water factories, with a detection frequency at 100% except for FF and SPD. Similarly, exclude SPD and FF, 23 chemicals were detected at a frequency higher than 60 (19 chemicals at 100%) in the wetland. This high detection frequency demonstrates ubiquitous of pharmaceuticals in the DWTP with both the conventional treatment process or the advanced treatment process and the wetland. The concentrations of pharmaceuticals were in the ranges of <LOD (FF, SPD) to 473.5 ng L^−1^ (ERY), <LOD (FF) to 404.7 ng L^−1^ (ERY), and <LOD to 59.5 ng L^−1^ (TYL) in DWTP A, DWTP B, and wetland C, respectively. ERY was observed the highest concentrations in both the waters factories and the wetland C, followed by PAR (27.3, 53.1, and 29.0 ng L^−1^ in DWTP A, DWTP B, and wetland C, respectively). Other 23 pharmaceuticals were all observed at low concentrations in the two factories and the wetland C (<20 ng L^−1^), except for FLE (25.8 ng L^−1^) and ENR (23.2 ng L^−1^) in DWTP B, as well as NOR and ENR (23.1 ng L^−1^) in wetland C. Highest concentrations of ERY and PAR were observed in water supply sources (DWTPs and the wetland) among all the 25 pharmaceuticals, which is in consistent to that in the surface water of Taihu Lake.

### Removal efficiencies

**Fig. 4:**
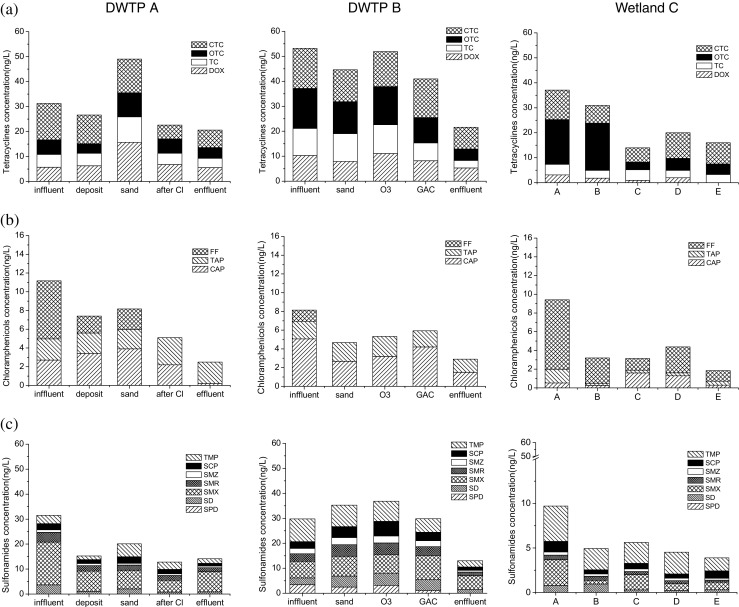

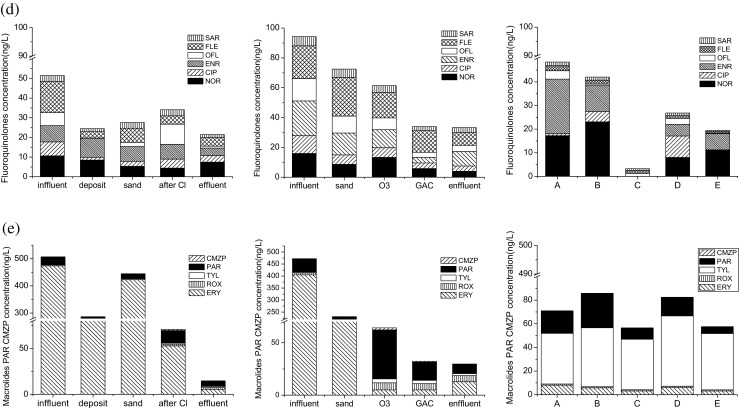
Concentrations of 25 pharmaceuticals in various compartments of the DWTP A, DWTP B, and wetland C. **a** Tetracyclines. **b** Chloramphenicols. **c** Sulfonamides and trimethoprim. **d** Fluoroquinolones. **e** Macrolides, paracetamol, and carbamazepine

The concentration variations of 25 pharmaceuticals in varied components of the two water factories and the wetland are shown in Fig. 4. In general, the concentrations decreased after various water treatment processes. Only two out of the 25 compounds (FF and SPD) were not detected in the effluent (finished water) of the two water factories, whereas four out of 25 compounds (DOX, SPD, CIP, and ROX) were not detected in the effluent (finished water) of wetland C. As shown in Table 2, the removal efficiencies were 2.9 to 100% in DWTP A with conventional water treatment process, 16.7 to 100% in DWTP B with advanced water treatment process, and 24.2 to 100% in wetland C with biological treatment processes. Except for sulfonamides, one chloramphenicol (CAP) and one macrolide (ROX), overall higher removal efficiencies were observed in B factor (advanced water treatment process) than those in DWTP A (conventional water treatment process). Low removal efficiencies (<50%) were observed for 10 out of 25 compounds in DWTP A, including 4 tetracyclines except for CTC (52.1%), 1 chloramphenicol (TAP 0), 1 sulfonamides (SMZ, 33.3%), TMP (44%), 2 fluoroquinolones (CIP 30.2%, SAR 46.7%), and 2 macrolides (ROX 49.1%, TYL 5.3%). Similarly, low removal efficiencies (<50%) were observed for 8 out of 25 compounds in DWTP B, including 2 tetracyclines (DOX 48.4%, CTC 45.6%), 1 chloramphenicols (TAP 25%), 2 sulfonamides (SD 45.1%, SMX 16.7%), 1 fluoroquinolone (SAR 43.5%), and 2 macrolides (ROX 31.8%, TYL 38.8%). However, only 6 out of 25 compounds were observed at low removal efficiencies (<50%) in wetland C, including 2 tetracyclines (TC 24.2%, CTC 28.4%), 1 chloramphenicol (CAP 43.3%), 2 sulfonamides (SMZ 42.4%, SCP 29.6), and 1 fluoroquinolone (NOR, 35%). The general good removal efficiencies for most pharmaceuticals by wetland C were consistent with previous research (Li et al. 2014) about removal of pharmaceuticals by constructed wetlands. Our results indicated that both the two water factories and the wetland can only efficiently remove a limited number of the 25 pharmaceuticals. In general, DWTP B with advanced treatment processes was superior to DWTP A with conventional treatment processes, except for sulfonamides. Wetland C could be used prior to the water factors in order to complement the water treatment processes in the water factories because high removal efficiencies were observed for different pharmaceuticals.

**Table 2:** The efficiencies of removal of pharmaceuticals in DWTPs and the wetland

Compounds		Removal efficiency (%)		
		DWTP A	DWTP B	Wetland C
Tetracyclines	DOX	2.9	48.4	100
	TC	27.3	72.4	24.2
	OTC	25.3	71.3	76.1
	CTC	52.1	45.6	28.4
Chloramphenicols	CAP	92.6	70.4	43.3
	TAP	0	25.0	71.4
	FF	100	100	84.8
Sulfonamides	SPD	–	100	–
	SD	76.4	45.1	61.7
	SMX	52.9	16.7	70.0
	SMR	59.3	51.1	56.4
	SMZ	33.3	67.2	42.4
	SCP	56.8	52.4	29.6
Trimethoprim	TMP	44.0	72.4	63.2
Fluoroquinolones	NOR	30.2	74.8	35.0
	CIP	52.1	70.8	100
	ENR	54.0	58.2	70.2
	OFL	85.9	72.7	94.6
	FLE	72.7	61.4	66.7
	SAR	46.7	43.5	74.1
Macrolides	ERY	98.8	96.8	61.7
	ROX	49.1	31.8	–
	TYL	5.3	38.8	–
Paracetamol	PAR	79.8	83.6	69.1
Carbamazepine	CMZP	64.3	86.0	59.0

*–* not available

In the conventional water treatment processes (DWTP A), coagulation/flocculation process (deposition) was very efficient for most of the compounds, especially good for sulfonamides and trimethoprim (total removal efficiency 43.48%), fluoroquinolones (total removal efficiency 56.72%), and macrolides (total removal efficiency 47.09%) (Fig. 4). However, the total removal efficiencies for tetracyclines (14.83%), chloramphenicols except FF, PAR, and CMZP were negligible (<15%). Coagulation/flocculation primarily aids in removing suspended solids (turbidity), high molecular weight natural organic matter, and micropollutants with log*K*ow >5. In this step, flocculant (aluminum sulfate) was added into the water sample and the suspended particles were flocculated and removed from the water phase. Some of the studied pharmaceuticals which are ionic at the current water pH may adsorb to particles and to the flocs formed in the coagulation by electrostatic interactions. Thus, the pharmaceuticals were removed simultaneously with the sedimentation. Good removal efficiency in our study was inconsistent to previous work, in which coagulation showed negligible effects on removal of pharmaceuticals (Vieno et al. 2007; Huerta-Fontela et al. 2011; Yan et al. 2015b). Low removal efficiency of coagulation/flocculation may be due to the lack of the suspended particulates in the raw water, and the removal could be improved by the particulate matter initially present in the water (Westerhoff et al. 2005). Thus, the suspended particulates in the raw water may improve the removal of pharmaceuticals. It was noteworthy to find that the concentrations of nearly all the compounds increased after sand filtration in DWTP A, especially significant increase for tetracyclines and ERY. The probable reason was that tetracyclines and ERY were absorbed by the sand and then desorbed. The final treatment step was based on an oxidation process with chlorineanie. It was found that chlorination was very efficient for nearly all the compounds except for sulfonamides. Previous work also demonstrated that various pharmaceuticals including macrolides, sulfonamides, hormones, and acid drugs like diclofenac (DIF) and naproxen were also efficiently removed by chlorination (Stackelberg et al. 2007). However, negligible removal of pharmaceuticals was observed in a recent work (Yan et al. 2015b), probably because the removal efficiency was influenced by the weather conditions, operational states of DWTPs, the sampling protocol, and the concentration levels of pharmaceuticals (Huerta-Fontela et al. 2011; Yan et al. 2015b).

In the advanced water treatment processes (DWTP B), ozonation with O_3_ and GAC adsorption were only efficient for fluoroquinolones, macrolides, PAR, and CMZP (Fig. 4d, e), which can well explain why general higher removal efficiencies for these compounds in DWTP B than these in DWTP A. This removal matches well to published results that ozonation and GAC were effective in removing pharmaceuticals (Cai et al. 2015). However, other compounds like tetracyclines, chloramphenicols, sulfonamides, and trimethoprim were removed mainly depending on traditional processes such as prechlorination and coagulation/flocculation in DWTP B. Conversely, concentrations of tetracyclines, chloramphenicols, sulfonamides, trimethoprim, and PAR increased after O_3_ (Fig. 4a–c). Similar concentration increases after O_3_ were also observed for several pharmaceuticals, such as bezafibrate, sulfamethoxazole, carbamazepine, and ibuprofen, in a recent work (Cai et al. 2015). In ozone treatment, ozone (O_3_) attacks the organic contaminants either by direct reaction (as molecular O_3_) or through the formation of free radicals, such as the hydroxyl radical (·OH), and usually results in the removal of organic compounds. The probably reason for concentration increase was that those compounds showed low reactivity with O_3_ (Cai et al. 2015). In addition, although the parent compounds of some pharmaceuticals can be effectively removed by ozonation, their possible more toxic transformation products should not be ignored. The GAC filtration processes are just transferring contaminants from a liquid phase to a solid phase, in which processes the hydrophobicity (log*K*ow) and molecule states (protonation or ionization) of the compounds in aquatic environment pH (Vieno et al. 2007) play a key role for the adsorption. Therefore, GAC filtration was found to result in an intermediate removal for several pharmaceuticals (carbamazepine andibuprofen) in literature (Cai et al. 2015), and it was not surprising to find that compounds like tetracyclines, chloramphenicols, sulfonamides, and trimethoprim in our study were not removed by GAC.

In the constructed wet land, the substrate, plants, and microbes are utmost important for understanding the removal mechanisms. Substrate, such as soil, gravel, and biosorbents (such as corn straws and *Kochia* straws in our study) is an important component in constructed wetlands, which not only provides support for the growth of plants and microorganisms but also interacts directly with contaminants through sorption processes. Sorption of pollutants onto the surface of substrate involves different mechanisms such as hydrophobic partitioning, van der Waals interaction, and electrostatic interaction. Nonpolar organic pollutants can be preferentially adsorbed to the substrate materials via hydrophobic interaction, whereas polar pollutants such as target pharmaceuticals in our study are dominantly adsorbed to the substrate materials by electrostatic interactions (Dordio and Carvalho 2013). Plants in constructed wetlands play a significant role in direct uptake of many organic pollutants from water. For pharmaceuticals, the uptake by plants is mainly driven by diffusion, in which process the physicochemical characteristics of pharmaceuticals such as hydrophobicity (log*K*ow) is of vital importance (Dordio and Carvalho 2013). Besides direct uptake, the internal pharmaceuticals might be degraded via the phytodegradation, including a series of biochemical reactions such as transformation of parent organic pollutants, conjugation of metabolites with macromolecules, and incorporation of conjugated products into plant cell walls and vacuoles. Plants in constructed wetlands also play another important role in stimulating the development and activities of microbial populations which are supported by the rhizodeposition products (exudates, mucigels, dead cell material), causing various biological processes to occur in the rhizosphere (Li et al. 2014). Microbes in constructed wetlands usually play the main role in the processes of transformation and mineralization of organic pollutants. It was reviewed (Li et al. 2014) that aerobic biodegradation was thought to be the main contribution to the microbial degradation process of ibuprofen, salicylic acid, and sulfamethoxazole, whereas anaerobic biodegradation was thought to be responsible for naproxen and caffeine. However, the degradation mechanisms of pharmaceuticals by microorganisms in constructed wetlands may also be influenced by the substrate, vegetation, oxygen and redox potential, temperature, pH, nutrient available, and presence of toxic substances, etc. Thus, it is still difficult to describe the actual removal mechanisms of pharmaceuticals in constructed wetlands to date.

Wetland C was very efficient for the removal of nearly all kinds of pharmaceuticals. Chloramphenicols (up to 84.8%) and sulfonamides (up to 70.0%) were mainly removed by the root channel wetland zone 1 (Fig. 4, process B), whereas other pharmaceuticals including tetracyclines, fluoroquinolones, ERY, PAR, and CMZP were mainly removed by the pump ascending and aeration aerobic zone (process C). In the root channel wetland zone 1 (process B), adsorption and biodegradation by plants, biosorbents, and soil contributed to the removal, suggesting that these process were effective for chloramphenicols and sulfonamides. However, in the pump ascending and aeration aerobic zone (process C), the organic compounds might be degraded by the complex processes including alternate redox interception, biodegradation, sorption by the solid, and synergy effects between physical, chemical, and biological approach of microorganisms. The prevalence of aerobic conditions in the wetland promotes biochemical pathways, such as aerobic respiration, that are more efficient in removing most emerging pollutants than anaerobic pathways (Matamoros et al. 2008). The fact that the mass of a pollutant in the effluent is lower than in the influent does not imply that the compound has been eliminated in the constructed wetland. Although it is true that some pollutants may have been degraded, transformed, or even mineralized in the constructed wetland, and their remains are expelled with the effluent, many others may have been retained as original compounds or as transformation products (TPs) in the wetland compartments (physical retention on the substrate or organic matter, adsorption onto biofilm or roots, incorporation to microbial biomass or to vegetal tissues, permanence in pore water).

## Conclusions

The overall contamination levels (0.2–74.9 ng L^−1^) of antibiotic pharmaceutics in the Taihu Lake were lower than or comparable to those reported worldwide. However, for nonantibiotic pharmaceutics, PAR (45.0 ng L^−1^) and CMZP (14.5 ng L^−1^), significantly higher concentrations were observed in the Taihu Lake in a global scale. High detection frequencies (>70%) were observed for nearly all the 25 pharmaceutics except for FF, SPD, ROX, and TYL. Tetracyclines (CTC max. 234.7 ng L^−1^), CAP (max. 27.1 ng L^−1^), ERY (max. 72.6 ng L^−1^), PAR (max. 71.7 ng L^−1^), and CMZP (max. 23.6 ng L^−1^) are compounds with both high a detection frequency (100%) and the highest concentrations, suggesting their wide use in the Taihu Basin. The detection frequency of pharmaceuticals in Taihu Lake is higher in dry season than that in wet season. However, an obvious seasonal variation trend (higher level in dry season) was only observed for chloramphenicols, macrolides, PAR, and CMZP, probably due to the low flow conditions of the lake in winter and the properties of pharmaceuticals. Besides, PAR is mainly used for curing a cold, of which the highest incidence is winter.

High detection frequencies of pharmaceuticals were detected in both the two water factories (100%) and the wetland (>60%, 19 chemicals at 100%) except for FF and SPD, demonstrating ubiquitous of pharmaceuticals in the DWTP with both the conventional treatment process or the advanced treatment process and the wetland. The highest concentrations of ERY (473.5 ng L^−1^) and PAR (53.1 ng L^−1^) were observed in water supply sources (DWTPs and the wetland) among all the 25 pharmaceuticals, which is in consistent to that in the surface water of Taihu Lake. Our results indicated that both the two water factories and the wetland can only efficiently remove a limited number of the 25 pharmaceuticals. In general, DWTP B with advanced treatment processes including ozonation and granular activated carbon filtration was superior to DWTP A with conventional treatment processes, except for sulfonamides. Wetland C could be used prior to the water factors in order to complement the water treatment processes in the water factories because higher removal efficiencies were observed for a variety of pharmaceuticals. Therefore, the application of cost-effective technologies such as constructed wetlands should be considered as an efficient alternative for reducing the amount of emerging contaminants such as pharmaceuticals from water supply sources. Further studies should be conducted to assess their efficiency as a polishing step of conventional sauce water treatments and in the removal of a wide range of pollutants such as pharmaceuticals.

## Electronic supplementary material

ESM 1 (DOCX 51 kb)
