# Supplementary material for: Occurrence of 25 pharmaceuticals in Taihu Lake and their removal from two urban drinking water treatment plants and a constructed wetland
Source: Environ Sci Pollut Res Int. 2017 May 6;24(17):14889–902. doi: 10.1007/s11356-017-8830-y (PMC6677712; doi:10.1007/s11356-017-8830-y)
Supplement: Supplementary file 1 — (DOCX 51 kb) [file 11356_2017_8830_MOESM1_ESM.docx]

**Supplementary Material**

Occurrence of 25 pharmaceuticals in Taihu Lake and their removal from two urban drinking water treatment plants and a constructed wetland

Xia-Lin Hu* • Yi-Fan Bao • Jun-Jian Hu • You-Yu Liu • Da-Qiang Yin

Key Laboratory of Yangtze River Water Environment, Ministry of Education, College of Environmental Science and Engineering, Tongji University, 1239 Siping Road, Shanghai, 200092, China.

*Corresponding author: Xia-Lin Hu

E-mail address: xlhu@tongji.edu.cn (Xia-Lin Hu)

Address:1239 Siping Road, Shanghai 200092, China.

Phone & Fax: +86-21-65982688.

Chemicals, sample preparation and analysis

All standards of pharmaceuticals and chemicals used in sample preparation and analysis were HPLC grade or analytical grade. Stock solutions of individual pharmaceuticals standards (200 mg L^-1^) were prepared and stored at -20℃. Working standard solutions were freshly prepared. Water samples were enriched by the solid-phase extraction (SPE) using the Oasis HLB cartridges (500 mg, 6 mL, Waters, Millford, MA, USA). Before extraction, water samples were filtered through 0.45 µm glass fiber filters (GF/F, Whatman, Mainstone, England), and then acidified to pH 3.0 with formic acid, followed by adding 0.5 g L^-1^ Na_2_EDTA as the chelating agent. Finally, isotopically labeled internal standards, including Sulfamethazine-^13^C_6_, Sulfamethoxazole-^13^C_6_, Ciprofloxacin (^13^C_3_^15^N), Erythrocin-^13^C_2,_ Trimethoprim-D_3_, were added in the samples with final concentrations at 25 ng L^-1^.

Prior to the extraction, Oasis HLB cartridges were conditioned successively with 6 mL of methanol, 6 mL of pure water (pH=3.0) at a flow rate of 1mL min^-1^. 1.0 L of each water sample was passed through the cartridge. Then, the cartridges were washed by 10 mL pure water to wipe off the remaining salt. The cartridges were dried with air under a fixed pressure to remove extra water for 10 to 20 min. In the end, cartridges were eluted by 3 mL methanol three times at a flow rate of 1 mL min^-1^ in a 10 mL centrifuge tube. The eluant was evaporated to dryness at room temperature under a gentle nitrogen stream and then reconstituted with 500 μL of methanol/water (1:4, v/v), the solution was then filtered through 0.22 μm PTFE filter before analysis by high performance liquid chromatography–mass spectrometry (UPLC-MS/MS, Waters Xevo TQ MS, Milford, MA, USA). All 25 pharmaceuticals were separated by a 150 mm BEH C_18_ column. Mass spectrometer detections of pharmaceuticals were operated in selected reaction monitoring mode. The details of separation and mass spectrometer parameters for each compound are described in Table S1 (Supplementary material).

Quantitative standard curve was obtained by plotting the ratio of the compound peak area with the internal standard peak area versus their concentrations. A series of mixed standard solutions were prepared (0, 0.1, 0.2, 0.5, 1, 2, 5, 10, 20, 50, 100, 200 ng L^-1^) to evaluate the linearity, at the same time 25 ng L^-1^ internal standards were added into water to counteract the loss of the targets in the pretreatment process to ensure the validity of the analysis results. The analytical performance of the SPE-HPLC-MS/MS method was satisfying for target compounds, with wide liner ranges (0.1–200 ng L^-1^) and good correlation coefficients (*R*^2^ > 0.99). The LODs and LOQs of 25 pharmaceuticals in surface water ranged from 0.12 to 2.24 ng L^-1^ and from 0.41 to 7.48 ng L^-1^, respectively. The recoveries of 25 pharmaceuticals (25 and 50 ng L^-1^) spiked to filtered surface water samples were mostly in the range of 70% to 110% (*n* = 3). The Intra-day relative standard deviation (RSD) (*n* = 3) and Inter-day RSD (*n* = 6) in the surface water was less than 15% and 25%, respectively. Analysis of reagent blanks demonstrated that the analytical system and glassware were free of contamination. The detailed information is shown in Table S2 (Supplementary material).

Ultra performance liquid chromatography-tandem mass spectrometry (UPLC-MS/MS) separation and mass spectrometer parameters

Waters ACQUITY UPLC system, equipped with a sample tray (4 ℃), autosampler, a binary pump and tandem Waters Xevo TQ MS, triple quadrupole mass spectrometer system with electrospray ionization (Electrospray Ionization Source, ESI) (Waters, Milford, Massachusetts, USA) was used for analysis. BEH C18 UPLC column (150 × 2.1 mm, 1.7μm., Waters) was used to separate 25 pharmaceuticals.

Liquid chromatography conditions were as follows：The column temperature was 40 ℃, the injection chamber temperature 5 ℃, the flow rate of 0.2 mL / min, the injection volume was 5µL. Gradient elution was employed .The mobile phase A was 0.1% formic acid/water, B was methanol, the water percentage was changed linearly as follows: 0 min: 80% A, 10.5 min: 10% A, 12min: 10% A, 12.1min: 80% A, 15min: 80% A. The whole analysis time was 15 min.

The main mass spectrometry conditions were as follows: the ionization voltage was 3.2kV, the cone voltage 35V, the desolvation temperature 150°C, the source temperature 120°C. Nitrogen (99.5% purity) was used as the desolvation gas at a flow of 550 L/Hr. High purity argon as the cone gas, the flow was at 0.13m L/min.

Among all the 25 pharmaceuticals, chloramphenicol antibiotics used the model of ESI -, others ESI+. Each individual pharmaceutical standard was directly injected into MS for automatic full scan with multiple reaction monitoring(MRM) to determine the relevant parameters including parent ions, quantitative ions, collision energy (CE). Detailed mass spectrum parameters were shown as follows (Table S1).

Table S1 Optimized Mass spectrum parameters of 25 pharmaceuticals

|  | Compounds | Abbreviation | Retention time(min) | Parent | Quantification | CV  (V) | CE (V) | Mode |
| --- | --- | --- | --- | --- | --- | --- | --- | --- |
| Tetracyclines | Doxycycline hyclate | DOX | 4.98 | 480.27 | 207.18 | 14 | 50 | ESI+ |
|  | Tetracycline hydrochloride | TC | 4.98 | 445.14 | 341.15 | 50 | 18 | ESI+ |
|  | Oxytetracycline hydrochloride | OTC | 5.08 | 461.17 | 429.28 | 62 | 12 | ESI+ |
|  | Chlortetracycline hydrochloride | CTC | 7.0 | 479.17 | 154.13 | 30 | 30 | ESI+ |
|  |  |  |  |  |  |  |  |  |
| Chloramphenicols | Chloramphenicol | CAP | 6.19 | 322.98 | 275.14 | 22 | 16 | ESI- |
|  | Thiamphenicol | TAP | 3.79 | 355.97 | 308.8 | 28 | 14 | ESI- |
|  | Florfenicol | FF | 4.78 | 357.97 | 241.11 | 30 | 20 | ESI- |
|  |  |  |  |  |  |  |  |  |
| Sulfonamides | Sulfapyridine | SPD | 3.23 | 266.96 | 251.12 | 100 | 24 | ESI+ |
|  | Sulfadiazine | SD | 2.85 | 250.96 | 156.1 | 30 | 16 | ESI+ |
|  | sulfamethoxazole | SMX | 2.85 | 253.96 | 156.11 | 26 | 16 | ESI+ |
|  | Sulfamerazine | SMR | 3.44 | 265.04 | 156.09 | 32 | 16 | ESI+ |
|  | Sulfamethazine | SMZ | 4.04 | 279.05 | 186.11 | 28 | 18 | ESI+ |
|  | Sulfachloropyridazine | SCP | 4.50 | 284.98 | 156.08 | 28 | 16 | ESI+ |
|  |  |  |  |  |  |  |  |  |
|  | Trimethoprim | TMP | 4.32 | 291.11 | 230.14 | 40 | 24 | ESI+ |
|  |  |  |  |  |  |  |  |  |
| Fluoroquinolones | Norfloxacin | NOR | 4.59 | 320.16 | 233.16 | 52 | 26 | ESI+ |
|  | Ciprofloxacin hydrochloride | CIP | 4.74 | 385.21 | 353.18 | 60 | 14 | ESI+ |
|  | Enrofloxacin | ENR | 4.78 | 360.2 | 245.21 | 40 | 28 | ESI+ |
|  | Ofloxacin | OFL | 4.37 | 362.17 | 261.22 | 38 | 30 | ESI+ |
|  | Fleroxacin | FLE | 4.15 | 370.1 | 269.15 | 34 | 28 | ESI+ |
|  | Sarafloxacin | SAR | 4.45 | 368.14 | 299.3 | 40 | 28 | ESI+ |
|  |  |  |  |  |  |  |  |  |
| Macrolides | Erythromycin | ERY | 10.89 | 734.65 | 158.21 | 32 | 28 | ESI+ |
|  | Roxithromycin | ROX | 6.85 | 837.75 | 158.21 | 34 | 32 | ESI+ |
|  | Tylosin Tartrate | TYL | 10.07 | 916.83 | 174.26 | 56 | 36 | ESI+ |
|  |  |  |  |  |  |  |  |  |
|  | Paracetamol | PAR | 2.87 | 152.99 | 71.06 | 24 | 6 | ESI+ |
|  | Carbamazepine | CMZP | 8.3 | 237.06 | 193.34 | 34 | 38 | ESI+ |

Table S2 LODs, LOQs, recoveries, RSD of 25 pharmaceuticals in surface water

| Compounds | Huangpu River | | | | | | | | Taihu Lake | | | | | | | |
| --- | --- | --- | --- | --- | --- | --- | --- | --- | --- | --- | --- | --- | --- | --- | --- | --- |
|  | LOD | LOQ | Recovery （%）± SD | | Inter RSD (%, *n* = 6) | | Intra RSD (%, *n* = 3) | | LOD | LOQ | Recovery （%）± SD | | Inter RSD (%, *n* = 6) | | Intra RSD (%, *n* = 3) | |
|  | （ng/L） | | 25 ng L^-1^ | 50 ng L^-1^ | 25 ng L^-1^ | 50 ng L^-1^ | 25 ng L^-1^ | 50 ng L^-1^ | ng L^-1^ | | 25 ng L^-1^ | 50 ng L^-1^ | 25 ng L^-1^ | 50 ng L^-1^ | 25 ng L^-1^ | 50 ng L^-1^ |
| DOX | 0.88 | 2.94 | 129.9±2.0 | 117.5±3.0 | 8 | 8 | 5 | 4 | 0.83 | 2.76 | 83.6±1.8 | 74.3±7.0 | 19 | 11 | 4 | 8 |
| TC | 0.44 | 1.47 | 108.3±1.0 | 102.4±3.0 | 6 | 5 | 5 | 2 | 0.77 | 2.58 | 92.1±0.3 | 79.4±7.3 | 25 | 8 | 1 | 8 |
| OTC | 1.00 | 3.33 | 124.2±1.7 | 107.7±1.6 | 5 | 2 | 3 | 1 | 1.30 | 4.32 | 90.2±1.5 | 79.3±1.5 | 7 | 9 | 3 | 2 |
| CTC | 0.84 | 2.79 | 99.3±3.8 | 104.2±1.0 | 5 | 3 | 2 | 3 | 0.99 | 3.31 | 82.9±1.7 | 77.3±7.9 | 8 | 7 | 3 | 9 |
| CAP | 0.96 | 3.21 | 87.7±1.2 | 74.8±0.8 | 7 | 9 | 1 | 4 | 2.24 | 7.48 | 82.0±2.2 | 75.5±5.9 | 6 | 4 | 5 | 7 |
| TAP | 1.01 | 3.37 | 103.9±1.0 | 92.6±1.6 | 2 | 3 | 3 | 4 | 0.41 | 1.37 | 84.1±3.0 | 77.9±4.7 | 5 | 9 | 7 | 6 |
| FF | 0.96 | 3.21 | 97.9±0.8 | 91.6±1.2 | 2 | 2 | 2 | 1 | 0.98 | 3.27 | 89.8±1.2 | 78.6±3.7 | 9 | 8 | 0 | 4 |
| SPD | 0.98 | 3.27 | 101.5±0.2 | 91.1±3.6 | 2 | 3 | 6 | 3 | 1.69 | 5.64 | 86.6±2.3 | 80.2±3.0 | 8 | 6 | 7 | 3 |
| SD | 0.40 | 1.33 | 101.8±1.8 | 91.6±2.4 | 1 | 1 | 4 | 2 | 1.07 | 3.58 | 87.1±1.5 | 73.3±4.4 | 10 | 13 | 3 | 5 |
| SMX | 0.75 | 2.51 | 90.3±1.2 | 101.4±0.8 | 5 | 5 | 1 | 3 | 0.86 | 2.88 | 74.3±0.3 | 78.2±2.0 | 13 | 7 | 5 | 2 |
| SMR | 0.23 | 0.75 | 102.3±1.0 | 101.3±3.6 | 1 | 1 | 7 | 0 | 0.90 | 3.02 | 89.9±1.0 | 72.4±4.9 | 10 | 20 | 3 | 6 |
| SMZ | 0.31 | 1.03 | 103.4±0 | 92.4±1.6 | 1 | 1 | 4 | 1 | 1.93 | 6.42 | 91.0±2.0 | 82.6±3.1 | 7 | 5 | 1 | 4 |
| SCP | 0.77 | 2.58 | 82.0±1.1 | 91.8±3.3 | 2 | 1 | 7 | 2 | 0.92 | 3.06 | 80.8±1.2 | 75.9±1.3 | 8 | 3 | 2 | 2 |
| TMP | 0.64 | 2.14 | 111.5±1.9 | 105.5±1.1 | 8 | 4 | 2 | 3 | 1.50 | 5.01 | 78.7±1.3 | 77.7±0.9 | 13 | 9 | 4 | 1 |
| NOR | 0.70 | 2.34 | 82.7±0.6 | 99.2±3.1 | 7 | 2 | 7 | 6 | 0.83 | 2.76 | 89.8±2.6 | 79.2±2.7 | 6 | 5 | 3 | 3 |
| CIP | 0.12 | 0.41 | 89.2±3.3 | 68.6±1.0 | 7 | 10 | 2 | 5 | 0.74 | 2.48 | 83.3±3.3 | 81.8±6.1 | 5 | 6 | 3 | 7 |
| ENR | 0.69 | 2.32 | 109.1±1.8 | 82.0±3.0 | 6 | 3 | 6 | 2 | 0.74 | 2.48 | 91.5±7.0 | 84.7±6.2 | 12 | 5 | 6 | 7 |
| OFL | 1.12 | 3.72 | 109.5±1.4 | 97.8±1.1 | 12 | 16 | 1 | 4 | 0.88 | 2.94 | 92.4±6.2 | 83.7±6.4 | 13 | 7 | 7 | 7 |
| FLE | 1.17 | 3.90 | 80.5±4.1 | 65.1±3.6 | 25 | 3 | 8 | 2 | 1.65 | 5.50 | 89.9±8.0 | 80.8±1.3 | 5 | 9 | 14 | 2 |
| SAR | 0.64 | 2.14 | 103.3±3.5 | 93.5±2.5 | 5 | 3 | 4 | 3 | 1.30 | 4.32 | 81.6±0.6 | 79.1±4.9 | 7 | 9 | 12 | 6 |
| ERY | 0.44 | 1.47 | 142.8±3.1 | 122.9±1.9 | 2 | 4 | 4 | 5 | 1.24 | 4.12 | 88.9±8.0 | 85.6±4.7 | 10 | 7 | 15 | 5 |
| ROX | 1.07 | 3.56 | 66.3±0.3 | 81.7±2.2 | 16 | 4 | 5 | 2 | 0.69 | 2.32 | 78.0±0.6 | 86.6±3.4 | 8 | 9 | 1 | 4 |
| TYL | 0.57 | 1.90 | 86.5±0.1 | 95.4±3.8 | 7 | 4 | 7 | 1 | 0.98 | 3.25 | 86.7±5.7 | 77.8±7.8 | 6 | 8 | 11 | 8 |
| PAR | 0.86 | 2.86 | 86.0±1.0 | 91.9±6.7 | 10 | 2 | 14 | 5 | 1.18 | 3.93 | 96.3±1.6 | 78.7±4.6 | 6 | 8 | 2 | 3 |
| CMZP | 0.80 | 2.66 | 104.0±0.1 | 93.5±1.4 | 5 | 4 | 3 | 3 | 0.84 | 2.79 | 91.8±1.1 | 86.6±2.0 | 9 | 6 | 2 | 2 |

Table S3 Detection frequency (*n* = 5 ) and concentrations of 25 pharmaceuticals in two drinking water treatment plants (DWTPs) and the wetland

| Compounds | | DWTP A | | | | | DWTP B | | | | | Wetland C | | | | | | | |
| --- | --- | --- | --- | --- | --- | --- | --- | --- | --- | --- | --- | --- | --- | --- | --- | --- | --- | --- | --- |
|  |  | Concentration (ng L^-1^) | | | | Frequency (%) | Concentration (ng L^-1^) | | | | Frequency (%) | Concentration (ng L^-1^) | | | | | | Frequency (%) | |
|  |  | Aver.^a^ | Med.^b^ | Max.^c^ | Min.^d^ |  | Aver.^a^ | Med.^b^ | Max.^c^ | Min.^d^ |  | Aver.^a^ | Med.^b^ | Max.^c^ | | Min.^d^ | |  |  |
| Tetracyclines | DOX | 8.0 | 6.3 | 15.7 | 5.5 | 100 | 8.6 | 8.2 | 11.1 | 5.3 | 100 | 1.9 | 1.9 | 3.1 | | <LOD | | 80 | |
|  | TC | 5.7 | 5.0 | 10.2 | 3.7 | 100 | 8.7 | 10.9 | 11.5 | 3.0 | 100 | 3.6 | 3.2 | 4.3 | | 3.0 | | 100 | |
|  | OTC | 5.9 | 5.7 | 9.7 | 3.9 | 100 | 11.7 | 12.7 | 16.0 | 4.6 | 100 | 9.8 | 4.8 | 18.9 | | 3.0 | | 100 | |
|  | CTC | 10 .4 | 11.5 | 14.6 | 5.5 | 100 | 13.4 | 14.0 | 16.1 | 8.7 | 100 | 8.7 | 8.4 | 11.8 | | 5.8 | | 100 | |
|  |  |  |  |  |  |  |  |  |  |  |  |  |  |  | |  | |  | |
| Chloramphenicols | CAP | 2.5 | 2.7 | 3.9 | 0.2 | 100 | 3.3 | 3.2 | 5.1 | 1.5 | 100 | 0.8 | 0.5 | 1.3 | | 0.3 | | 100 | |
|  | TAP | 2.3 | 2.3 | 2.1 | 2.9 | 100 | 1.8 | 1.9 | 2.1 | 1.4 | 100 | 0.5 | 0.3 | 1.5 | 0.2 | | 100 | |  |
|  | FF | 3.4 | 2.2 | 6.2 | <LOD^e^ | 60 | 1.2 | <LOD | 1.2 | <LOD | 20 | 3.1 | 2.7 | 7.4 | | 1.1 | | 100 | |
|  |  |  |  |  |  |  |  |  |  |  |  |  |  |  | |  | |  | |
| Sulfonamides | SPD | <LOD | <LOD | <LOD | <LOD | 0 | 2.5 | 3.1 | 3.4 | <LOD | 80 | <LOD | <LOD | <LOD | | <LOD | | 0 | |
|  | SD | 1.7 | 1.1 | 3.7 | 0.8 | 100 | 3.6 | 4.4 | 4.7 | 1.5 | 100 | 0.5 | 0.3 | 0.9 | | 0.2 | | 100 | |
|  | SMX | 9.1 | 8.1 | 17.1 | 4.7 | 100 | 7.5 | 7.6 | 9.7 | 5.5 | 100 | 1.3 | 0.9 | 2.9 | | 0.4 | | 100 | |
|  | SMR | 2.3 | 2.1 | 3.9 | 1.6 | 100 | 3.5 | 3.6 | 4.7 | 1.5 | 100 | 0.4 | 0.3 | 0.5 | | 0.2 | | 100 | |
|  | SMZ | 0.8 | 0.7 | 1.0 | 0.7 | 100 | 2.2 | 2.4 | 2.9 | 0.7 | 100 | 0.3 | 0.3 | 0.4 | | 0.2 | | 100 | |
|  | SCP | 1.9 | 1.7 | 2.6 | 1.1 | 100 | 3.5 | 3.3 | 5.9 | 1.3 | 100 | 0.7 | 0.6 | 1.2 | | 0.5 | | 100 | |
|  |  |  |  |  |  |  |  |  |  |  |  |  |  |  | |  | |  | |
| Trimethoprim | TMP | 3.0 | 2.9 | 5.2 | 1.5 | 100 | 6.7 | 8.1 | 9.1 | 2.5 | 100 | 2.5 | 2.4 | 4.0 | | 1.5 | | 100 | |
|  |  |  |  |  |  |  |  |  |  |  |  |  |  |  | |  | |  | |
| Fluoroquinolones | NOR | 7.2 | 7.4 | 10.6 | 4.4 | 100 | 9.5 | 8.7 | 15.9 | 4.0 | 100 | 14.9 | 11.2 | 23.1 | | <LOD | | 80 | |
|  | CIP | 3.8 | 3.4 | 7.1 | 1.4 | 100 | 6.4 | 6.3 | 12.0 | 3.5 | 100 | 4.8 | 0.8 | 9.1 | | <LOD | | 60 | |
|  | ENR | 7.4 | 7.7 | 9.7 | 3.8 | 100 | 12.7 | 12.1 | 23.2 | 3.7 | 100 | 11.5 | 6.9 | 23.1 | | <LOD | | 80 | |
|  | OFL | 4.0 | 2.1 | 6.6 | 0.1 | 100 | 8.4 | 7.9 | 15.0 | 3.5 | 100 | 1.6 | 1.2 | 3.5 | | 0.2 | | 100 | |
|  | FLE | 7.0 | 4.3 | 15.9 | 3.3 | 100 | 17.6 | 17.0 | 25.8 | 25.8 | 100 | 1.4 | 1.3 | 2.2 | | 0.7 | | 100 | |
|  | SAR | 2.4 | 2.9 | 3.1 | 1.5 | 100 | 4.6 | 4.7 | 6.2 | 2.7 | 100 | 1.0 | 1.0 | 1.6 | | 0.4 | | 100 | |
|  |  |  |  |  |  |  |  |  |  |  |  |  |  |  | |  | |  | |
| Macrolides | ERY | 240.9 | 250.0 | 473.5 | 5.5 | 100 | 122.2 | 12.8 | 404.7 | 5.1 | 100 | 5.3 | 5.7 | 8.1 | | 3.1 | | 100 | |
|  | ROX | 2.7 | 2.0 | 1.9 | 3.7 | 100 | 6.8 | 6.7 | 8.5 | 5.8 | 100 | <LOD | <LOD | <LOD | | <LOD | | 0 | |
|  | TYL | 1.2 | 1.2 | 1.2 | 1.3 | 100 | 3.7 | 3.3 | 6.2 | 2.0 | 100 | 48.6 | 47.6 | 59.5 | | 42.9 | | 100 | |
|  |  |  |  |  |  |  |  |  |  |  |  |  |  |  | |  | |  | |
| Paracetamol | PAR | 18.9 | 16.1 | 27.3 | 5.5 | 100 | 31.6 | 31.8 | 53.1 | 8.7 | 100 | 15.7 | 15.5 | 29.0 | | 5.8 | | 100 | |
|  |  |  |  |  |  |  |  |  |  |  |  |  |  |  | |  | |  | |
| Carbamazepine | CMZP | 1.1 | 1.3 | 1.4 | 0.5 | 100 | 1.8 | 1.9 | 3.1 | 0.4 | 100 | 0.2 | 0.2 | 0.2 | | 0.1 | | 100 | |

a Average

b Median

c Maximum

d Minimal

e LOD: limit of detection


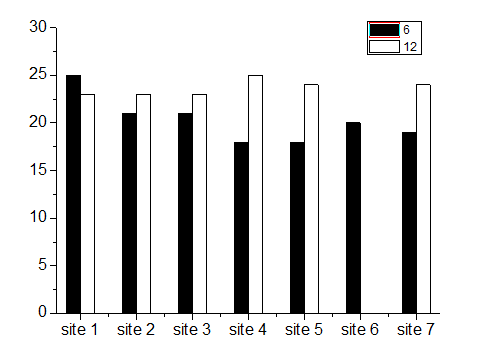


**Fig. S1** The number of detected compounds in all the sampling sites in June (6) and December (12).
